# Digital Competence and Career Adaptability Among Nurses: The Parallel Mediating Roles of Technological Self‐Efficacy and Learning Agility

**DOI:** 10.1155/jonm/7770229

**Published:** 2026-04-17

**Authors:** Ibrahim Abdullatif Ibrahim, Samar Atef Mounib, Eman Saad Eldesoky, Reda Shehata Elsayed, Mennat Allah G. Abou Zeid, Hala Gaber Elatroush

**Affiliations:** ^1^ Department of Nursing Sciences, College of Applied Medical Sciences, Shaqra University, Shaqra, Saudi Arabia, su.edu.sa; ^2^ Nursing Administration Department, Faculty of Nursing, Mansoura University, Mansoura, Egypt, mans.edu.eg; ^3^ Nursing Administration Department, Faculty of Nursing, Beni-suef University, Beni-suef, Egypt, bsu.edu.eg; ^4^ Nursing Department, Al-Ghad College for Applied Medical Sciences, Dammam, Saudi Arabia; ^5^ Nursing Administration and Education Department, College of Nursing, Prince Sattam Bin Abdulaziz University, Al-Kharj, Saudi Arabia, psau.edu.sa; ^6^ Department of Nursing Administration, Faculty of Nursing, Ain Shams University, Cairo, Egypt, asu.edu.eg

**Keywords:** career mobility, learning, nursing, professional competence, self-efficacy, structural equation modeling, technology

## Abstract

**Background:**

The rapid digital transformation of healthcare systems requires nurses to continuously update their competencies to remain effective, adaptable, and resilient. Although digital competence, technological self‐efficacy, and learning agility are recognized as key factors influencing career adaptability, the mechanisms connecting these constructs within nursing contexts remain underexplored.

**Aim:**

This study investigated the relationship between digital competence and career adaptability among clinical nurses, with a focus on the mediating roles of technological self‐efficacy and learning agility.

**Methods:**

A cross‐sectional, correlational design was employed, involving 307 nurses recruited from two specialized university hospitals using stratified sampling. Data were collected using validated instruments measuring digital competence, technological self‐efficacy, learning agility, and career adaptability. Partial Least Squares Structural Equation modeling was utilized to test the hypothesized parallel mediation model, following STROBE reporting guidelines.

**Results:**

Nurses reported a moderate level of digital competence (*M* = 3.46, SD = 0.42) and technological self‐efficacy (*M* = 3.64, SD = 0.46), while learning agility and career adaptability were rated high (*M* = 3.85, SD = 0.47; *M* = 4.50, SD = 0.43, respectively). Digital competence had significant positive direct effects on career adaptability (*β* = 0.185, *p* < 0.01), learning agility (*β* = 0.184, *p* < 0.01), and technological self‐efficacy (*β* = 0.254, *p* < 0.001). Both learning agility (*β* = 0.167, *p* < 0.01) and technological self‐efficacy (*β* = 0.236, *p* < 0.001) were also significant predictors of career adaptability. Moreover, digital competence exerted significant indirect effects on career adaptability through technological self‐efficacy (*β* = 0.060, *p* < 0.05) and learning agility (*β* = 0.031, *p* < 0.05).

**Conclusion:**

Digital competence emerged as a fundamental determinant of nurses’ career adaptability, operating directly and indirectly through technological self‐efficacy and learning agility. These findings highlight the importance of fostering digital readiness and adaptive learning among nurses. Nursing leaders should prioritize professional development initiatives to enhance workforce adaptability and sustain effective practice in technology‐driven healthcare environments.

## 1. Introduction

The healthcare sector has experienced rapid digital transformation, reshaping nursing roles, responsibilities, and required competencies [1]. Technologies such as electronic health records, telehealth platforms, and clinical decision‐support systems are now integral to patient care and hospital operations [2]. In this evolving environment, digital competence is no longer optional; it encompasses not only the technical ability to use digital tools but also the confidence, critical thinking, and ethical awareness needed to apply them effectively in clinical practice [3].

Evidence shows that digital competence is increasingly emphasized in nursing education and clinical practice, yet structured programs for practicing nurses remain limited [4, 5]. Simultaneously, career adaptability has become vital in technology‐driven healthcare settings. Nurses with higher career adaptability are better prepared to adopt new systems, adjust workflows, and respond to evolving professional demands [6].

Two psychological mechanisms are particularly relevant in this context. Technological self‐efficacy reflects confidence in one’s ability to successfully carry out digital and technological tasks, influencing readiness to meet healthcare challenges and supporting the overall digital advancement of healthcare practices [7, 8]. Learning agility represents the ability to learn from experience and apply knowledge in new situations, supporting professional adaptability and performance in dynamic clinical environments [9, 10]. Both technological self‐efficacy and learning agility have been linked to effective adaptation and performance in organizational and educational contexts, yet empirical evidence in practicing nurses remains limited.

Despite the growing recognition of digital competence, technological self‐efficacy, and learning agility, there is a paucity of research investigating how digital competence contributes to nurses’ career adaptability and through which mechanisms this relationship operates in real‐world clinical settings. Addressing this gap is essential for informing workforce development, enhancing digital readiness, and fostering professional growth among nurses. Consequently, this study contributes valuable insights into the relationship between digital competence and career adaptability, informing strategies to support nurses in enhancing their technological skills, self‐assurance, and adaptability within dynamic healthcare settings.

## 2. Background

### 2.1. Digital Competence in Nursing

Digital competence refers to knowledge, skills, attitudes, and strategies that enable individuals to effectively navigate new technological situations, critically analyze and evaluate information, solve problems using technology, and collaborate in digital environments [11, 12]. These competencies are essential for lifelong learning and for maintaining professional adaptability in the rapidly evolving healthcare environment [13, 14]. Nurses with higher levels of digital competence are more familiar with digital interfaces, more efficient in performing tasks, and more confident in managing complex technological systems [15]. On the other side, nurses who lack digital competence may struggle to manage technological systems effectively, which can limit both organizational performance and patient outcomes [16].

### 2.2. Digital Competence and Career Adaptability

Career adaptability reflects nurses’ readiness and resources to navigate vocational challenges, role transitions, and unpredictable professional events. It encompasses concern, control, curiosity, and confidence, which enable professionals to respond effectively to career demands [17]. Nurses who adapt to their career are more effective and have less turnover and high productivity. Nurses with strong digital competence are better positioned to leverage technology in managing daily tasks, making informed decisions, and addressing complex clinical and administrative challenges [18]. By effectively integrating digital tools into their work, they can enhance problem‐solving, improve workflow efficiency, and maintain high standards of care [19]. Consequently, digital competence directly supports nurses’ ability to adapt to changing career environments and sustain professional effectiveness [1].

### 2.3. The Mediating Role of Technological Self‐Efficacy

Technological self‐efficacy refers to nurses’ confidence in their ability to use digital technologies effectively and to adapt to technological changes [20, 21]. High technological self‐efficacy reduces technology‐related anxiety, promotes persistence, and enhances the quality of task performance [22, 23]. Research consistently shows a positive, mutually reinforcing relationship between digital competence and technological self‐efficacy among nurses and nursing students [24, 25]. Higher digital competence enhances self‐efficacy, and greater self‐efficacy encourages the development and application of digital skills [26]. Moreover, nurses with strong digital competence tend to develop higher technological self‐efficacy, which in turn allows them to leverage digital tools more effectively and respond confidently to challenges in the workplace [24]. Importantly, technological self‐efficacy serves as a mechanism through which digital competence influences career adaptability [27]. Consequently, nurses who feel confident in their technological skills are better able to apply these skills to professional challenges, enhancing their adaptability in dynamic clinical and organizational contexts.

### 2.4. The Mediating Role of Learning Agility

Learning agility is defined as the willingness and ability to learn from experience and apply acquired knowledge to novel situations for successful performance [28]. It encompasses motivation, adaptability, and the potential to acquire new competencies, which are crucial for professional growth.

Digital competence significantly predicts learning agility outcomes (e.g., academic motivation and clinical decision‐making) in nursing students and professionals. For example, Amin et al. [29] found that nursing students’ digital competence strongly predicted academic motivation and lifelong learning tendencies, and Patwardhan et al. [30] observed a positive relationship between perceived digital competence and learning agility among students. Moreover, learning agility mediated the relationship between perceived digital competence and perceived learning outcomes.

Digital competence may promote learning agility among nurses by enabling effective use of technological resources for information retrieval, knowledge acquisition, and adaptation to rapidly changing clinical and organizational environments. Nadzim et al. [31] showed that digital competence enhances employee agility, and empowerment was found to strengthen the relationship between information and data literacy and employee agility.

Learning agility also acts as a mediator between digital competence and career adaptability. Nurses with high learning agility can apply their digital skills flexibly, learn from experience, and respond effectively to unpredictable career challenges, thereby enhancing their adaptability and overall professional outcomes. A recent review suggests digital competence and new technology adaptability enhance learning agility, which in turn relates to work engagement and performance [32].

### 2.5. Study Aim and Hypotheses

Building on the theoretical relationships outlined above, this study aimed to examine the relationship between digital competence and career adaptability among nurses in technology‐intensive healthcare environments. Specifically, the study investigated whether technological self‐efficacy and learning agility operate as parallel mediators in this relationship.

Based on the literature, the following hypotheses were proposed:

 H1: Digital competence positively predicts career adaptability.
 H2: Digital competence positively predicts technological self‐efficacy.
 H3: Technological self‐efficacy mediates the relationship between digital competence and career adaptability.
 H4: Digital competence positively predicts learning agility.
 H5: Learning agility mediates the relationship between digital competence and career adaptability.

## 3. Methods

### 3.1. Design

This study employed a cross‐sectional, correlational design and was reported in accordance with the STROBE guidelines, ensuring transparent, complete, and standardized documentation of the observational research.

### 3.2. Setting and Participants

The study was conducted in two specialized university hospitals affiliated with Mansoura University in Egypt: Mansoura University Children’s Hospital (MUCH) and Oncology Center Mansoura University (OCMU). MUCH is a large tertiary hospital that provides specialized pediatric care, while OCMU is one of the country’s leading oncology centers, offering comprehensive services for oncology patients. These hospitals were selected because they are highly digitalized, employ nurses with diverse backgrounds, and actively use technology in daily patient care. Such features made them suitable environments for studying how nurses’ digital competence, confidence with technology, and learning agility relate to their ability to adapt in their careers.

The study targeted nurses working in various clinical units within the two selected hospitals. Eligible participants were those who had a minimum of 6 months of clinical experience and provided informed consent to take part in the study. Nurses assigned to administrative duties or on extended leave during the data collection period were excluded. A stratified sampling approach was used to recruit participants.

The required sample size was determined using the a priori Sample Size Calculator for Structural Equation Modelling (SEM), assuming a medium effect size (*f* ^2^ = 0.3), a significance level of 0.05, and a statistical power of 0.95. With four latent variables and 43 observed variables, the minimum sample size was estimated at 207 nurses. To ensure robustness and enhance the generalizability of the findings, 342 nurses were invited to participate, of whom 307 returned valid and complete questionnaires, resulting in a response rate of 89.8%. The remaining 35 nurses (10.2%) declined to participate in the study.

### 3.3. Data Collection Procedure

Data were collected between August and October 2025 using a structured, self‐administered paper‐based questionnaire distributed to nurses in the predetermined settings. To minimize missing data and ensure completeness, the questionnaires were designed with clear instructions and consistent response formats. The contact information of the third and fourth coauthors was listed on the cover page for any inquiries related to the questionnaire and coordinated its distribution and collection. Questionnaires were distributed across different shifts and units. The coauthors remained available when needed to ensure completion, and completed forms were reviewed immediately. Participants were prompted to fill in any missing items. For nurses unable to complete the questionnaire immediately, follow‐up arrangements were made to collect the remaining responses. These procedures effectively prevented missing data. The time required to complete the questionnaire ranged from 10 to 15 min, and only fully completed questionnaires were included in the final analysis to maintain data quality and reliability.

### 3.4. Instruments

Data were collected using a structured, self‐administered questionnaire composed of five sections (see Table 1s) as described below.

#### 3.4.1. Section 1: Demographic Information

This section gathered information about participants’ personal and professional characteristics, including age, gender, marital status, educational level, and years of experience.

#### 3.4.2. Section 2: Digital Competence Questionnaire (DCQ)

The DCQ was developed and validated by Golz et al. [33] was employed to measure perceived levels of digital competence among nurses. The instrument comprises 12 items distributed across two dimensions: knowledge and skills (6 items) and attitude (6 items). Items are rated on a five‐point Likert scale ranging from 1 (strongly disagree) to 5 (strongly agree). Higher scores indicate greater levels of digital competence.

#### 3.4.3. Section 3: Digital and Technological Self‐Efficacy Scale (Digitech‐S)

The Digitech‐S, developed by Conte et al. [8], was used to assess nurses’ confidence in performing digital and technology‐related tasks. The scale consists of 10 items covering a wide range of digital capabilities, including online communication, e‐learning, and digital problem‐solving. Responses are provided on a five‐point Likert scale (1 = strongly disagree to 5 = strongly agree), with higher scores reflecting stronger technological self‐efficacy.

#### 3.4.4. Section 4: Learning Agility Scale (LAS)

Learning agility was measured using a nine‐item unidimensional LAS, adapted from Bedford [34]. This scale was used to assess nurses’ ability to learn from experience and adapt to new situations in the workplace. Each item is rated on a five‐point Likert scale ranging from 1 (strongly disagree) to 5 (strongly agree). Higher mean scores reflect greater learning agility.

#### 3.4.5. Section 5: Career Adapt‐Abilities Scale–Short Form (CAAS‐SF)

Career adaptability was assessed using the CAAS‐SF, developed by Maggiori et al. [17]. The instrument includes 12 items equally divided into four subscales: Concern, Control, Curiosity, and Confidence. Each item is rated on a five‐point Likert scale ranging from 1 (never) to 5 (always). Higher total and subscale scores indicate stronger career adaptability resources.

All study instruments were first translated from English into Arabic and then back‐translated to English following Beaton et al.’s guidelines to ensure both linguistic accuracy and cultural appropriateness [35]. A panel of five experts reviewed the translations to confirm clarity and content validity. A pilot test with 19 nurses assessed the clarity and feasibility of the questionnaire, and these data were excluded from the main analysis. Based on feedback, the CAAS‐SF was modified from a 5‐point scale ranging from 1 (“not a strength”) to 5 (“greatest strength”) to a 5‐point scale from 1 (“never”) to 5 (“always”), and the LAS was adapted from its original 7‐point scale to a 5‐point scale (1 = strongly disagree to 5 = strongly agree). These changes simplified response options, improved clarity, and strengthened the reliability of the measures.

### 3.5. Data Analysis

The study employed a two‐pronged approach for data analysis. Initially, descriptive statistics were conducted using IBM SPSS Statistics version 27 to summarize the participants’ demographic characteristics and the study variables. Measures included frequencies, percentages, means, and standard deviations, providing a clear overview of the sample profile and variable distributions.

For the hypotheses testing and structural model assessment, Partial Least Squares SEM (PLS‐SEM) was applied using SmartPLS 4.0. Given the presence of higher‐order constructs, a disjoint two‐stage approach was adopted, following established recommendations for reflective–reflective hierarchical component models [36].

In the first stage, the lower‐order constructs (LOCs) were analyzed to assess measurement model properties, including indicator loadings, internal consistency reliability (Cronbach’s alpha and Composite Reliability, CR), convergent validity (Average Variance Extracted, AVE), and multicollinearity (Variance Inflation Factor, VIF). Discriminant validity was evaluated using the Heterotrait–Monotrait (HTMT) ratio and the Fornell–Larcker criterion. This stage ensured that all LOCs were valid and reliable before computing scores for higher‐order constructs (HOCs) (see supporting file Tables 2s‐3s).

In the second stage, the latent variable scores obtained from the first stage were used to model the HOCs. The measurement model based on these latent scores was then assessed to ensure that the HOCs maintained adequate reliability, convergent validity, and discriminant validity before proceeding to evaluate the structural relationships.

The structural model was then evaluated to examine hypothesized relationships among constructs. Path coefficients (β), significance values (t‐statistics and *p* values), coefficient of determination (*R* ^2^), effect sizes (*f* ^2^), predictive relevance (*Q* ^2^), and model fit indices including Standardized Root Mean Square Residual (SRMR), d_ULS, d_G, chi‐square, and Normed Fit Index (NFI) were estimated. Bootstrapping with 5000 resamples was applied to test direct, indirect, and total effects, including mediation pathways. By employing this disjointed two‐stage approach, the study ensured accurate estimation of the hierarchical model while maintaining rigorous assessment of both measurement and structural properties. This method provides robust and unbiased parameter estimates for complex constructs and their interrelationships.

### 3.6. Ethical Considerations

Ethical approval for this study was obtained from the Institutional Ethical Research Committee at the Nursing Faculty, Mansoura University, Egypt (Approval No: 0809) before data collection began. Permission to conduct the study was also granted orally by the Director of Nursing and the head nurses at the participating hospitals. The study adhered to the ethical principles of the Declaration of Helsinki. All participants were clearly informed about the purpose of the study, that their participation was voluntary, and that they could withdraw at any time without any consequences. A written informed consent form was provided at the start of the survey. To protect anonymity and maintain confidentiality, no identifying information was collected, and all responses were stored securely with access restricted to the research team.

## 4. Results

### 4.1. Participant Demographics

The study included a total of 307 nurses whose ages, with a mean of 31.40 years (SD = 7.91). More than half of the respondents (57.0%) were younger than 30 years, 30.6% were aged 31–40 years, and only 12.4% were older than 40. The sample was predominantly female (78.8%), with male nurses representing 21.2%. Regarding marital status, more than half of the respondents (55.0%) were married, held a technical degree (57.7%), and had 1–5 years of experience (60.3%) Table 1.

**TABLE 1:** Demographic information of participants.

Characteristics	*N*	%
*Age (years)*		
< 30	175	57.0
31–40	94	30.6
> 40	38	12.4
Mean (SD)	31.40 (7.91)	

*Gender*		
Male	65	21.2
Female	242	78.8

*Marital status*		
Unmarried	138	45.0
Married	169	55.0

*Education*		
Technical degree	177	57.7
Bachelor degree	130	42.3

*Experience*		
1–5	185	60.3
6–10	53	17.3
> 10	69	22.5

Regarding the study variables, nurses reported a moderate level of digital competence (*M* = 3.46, SD = 0.42), with its dimensions, knowledge and skills (*M* = 3.50, SD = 0.44) and attitude (*M* = 3.42, SD = 0.51), showing similar moderate levels. Technological self‐efficacy was also rated at a moderate level (*M* = 3.64, SD = 0.46), while learning agility was reported at a high level (*M* = 3.85, SD = 0.47). Career adaptability demonstrated a high level overall (*M* = 4.50, SD = 0.43), with its subdimensions; concern (*M* = 4.47, SD = 0.38), control (*M* = 4.53, SD = 0.46), confidence (*M* = 4.52, SD = 0.48), and curiosity (*M* = 4.47, SD = 0.49), all reflecting consistently high levels (see supporting file Table 4s).

### 4.2. Measurement Model: Reliability and Validity

Table 2 presents the reliability and convergent validity of the study constructs. All constructs demonstrated acceptable psychometric properties. For digital competence, both dimensions (knowledge and skills and attitude) had high loadings (0.858–0.905), with satisfactory internal consistency (*α* = 0.716, CR = 0.875) and good convergent validity (AVE = 0.778). The technological self‐efficacy construct showed strong loadings ranging from 0.731 to 0.803, excellent reliability (*α* = 0.927, CR = 0.938), and adequate convergent validity (AVE = 0.601). Similarly, learning agility recorded high item loadings (0.736–0.784), strong reliability (*α* = 0.910, CR = 0.924), and acceptable validity (AVE = 0.575). For career adaptability, all four dimensions loaded strongly (0.763–0.833), with satisfactory reliability (*α* = 0.800, CR = 0.868) and convergent validity (AVE = 0.621). Following the full collinearity approach recommended for PLS‐SEM, all VIF values were below the conservative threshold of 3.3, indicating that common method bias is unlikely to pose a serious threat to the validity of the findings.

**TABLE 2:** Loadings, collinearity statistics, reliability, and convergent validity.

Constructs	Dimensions/items	Loading	Collinearity statistics	Reliability		Convergent validity
			VIF	α	CR	AVE
DC	Knowledge and Skills	0.905	1.452	0.716	0.875	0.778
	Attitude	0.858	1.452			

TSE	TSE1	0.731	2.366	0.927	0.938	0.601
	TSE2	0.749	2.355			
	TSE3	0.769	2.577			
	TSE4	0.772	2.582			
	TSE5	0.779	2.247			
	TSE6	0.797	2.296			
	TSE7	0.803	2.417			
	TSE8	0.759	2.125			
	TSE9	0.803	2.491			
	TSE10	0.791	2.454			

LA	LA1	0.765	1.846	0.910	0.924	0.575
	LA2	0.764	2.018			
	LA3	0.744	1.810			
	LA4	0.764	1.906			
	LA5	0.784	2.027			
	LA6	0.736	2.440			
	LA7	0.759	2.503			
	LA8	0.764	2.503			
	LA9	0.740	2.251			

CA	Concern	0.833	1.629	0.800	0.868	0.621
	Control	0.776	1.760			
	Confidence	0.763	1.557			
	Curiosity	0.778	1.793			

Abbreviations: CA = career adaptability, DC = digital competence, LA = learning agility, TSE = technological self‐efficacy.

### 4.3. Discriminant Validity

Discriminant validity was confirmed using the HTMT ratio, the Fornell–Larcker criterion, and cross‐loadings. The HTMT values ranged from 0.199 to 0.362, all well below the recommended threshold of 0.85, indicating satisfactory discriminant validity among the study constructs. Consistent with this, the Fornell–Larcker criterion further confirmed discriminant validity, as the square roots of the AVE values (displayed on the diagonal) were greater than the corresponding inter‐construct correlations. Specifically, digital competence (0.882), technological self‐efficacy (0.776), learning agility (0.758), and career adaptability (0.788); each construct demonstrated stronger associations with their respective indicators than with other constructs. These results collectively affirm that the measurement model possesses adequate discriminant validity, ensuring that each construct represents a distinct dimension of the conceptual framework (Table 3).

**TABLE 3:** Discriminant validity assessment.

HTMT criterion	DC	TSE	LA	CA
*DC*				
TSE	0.291			
LA	0.199	0.229		
CA	0.362	0.345	0.276	

*Fornell*–*Larcker criterion*				
DC	0.882			
TSE	0.254	0.776		
LA	0.184	0.232	0.758	
CA	0.275	0.321	0.255	0.788

Abbreviations: CA = career adaptability, DC = digital competence, LA = learning agility, TSE = technological self‐efficacy.

### 4.4. Structural Model Evaluation

Table 4 presents the key metrics for assessing the structural model, including the coefficients of determination (*R* ^2^), effect sizes (*f* ^2^), predictive relevance (*Q* ^2^), and model fit indices. The *R* ^2^ values indicate that digital competence, technological self‐efficacy, and learning agility collectively explained 16.9% of the variance in career adaptability, 3.4% in learning agility, and 6.5% in technological self‐efficacy, suggesting that the model accounts for a modest yet meaningful proportion of variance in the endogenous constructs.

**TABLE 4:** Structural model metrics.

Constructs	*R*‐square	R‐square adjusted	*f*‐square	*Q* ^2^ predict	VIF	Model fit indices	
DC‐ > CA			0.038		1.088	SRMR	0.071
DC‐ > LA			0.035		1.000	d_ULS	2.192
DC‐ > TSE			0.069		1.000	d_G	0.507
LA‐ > CA			0.031		1.076	Chi‐square	907.521
TSE‐ > CA			0.060		1.111	NFI	0.791
CA	0.169	0.161		0.066			
LA	0.034	0.031		0.022			
TSE	0.065	0.062		0.052			

Abbreviations: CA = career adaptability, DC = digital competence, LA = learning agility, TSE = technological self‐efficacy.

The *f* ^2^ values ranged from 0.031 to 0.069, reflecting small to moderate effect sizes according to Cohen’s criteria [37]. Therefore, these effect sizes should be interpreted cautiously. The *Q* ^2^ values (0.022–0.066) were all positive, demonstrating acceptable predictive relevance of the model. The VIF values, which ranged from 1.000 to 1.111, were well below the cutoff of 5, confirming the absence of multicollinearity. Furthermore, the SRMR value of 0.071 indicates an acceptable model fit, as it is below the recommended threshold of 0.08. Additional fit indices also support the adequacy of the model, including d_ULS = 2.192, d_*G* = 0.507, chi‐square = 907.521, and NFI = 0.791. Overall, these results suggest that the structural model demonstrates acceptable explanatory power, predictive relevance, and model fit, providing a sound basis for hypothesis testing.

### 4.5. Structural Model (Direct, Indirect, and Total Effects)

**FIGURE 1:**
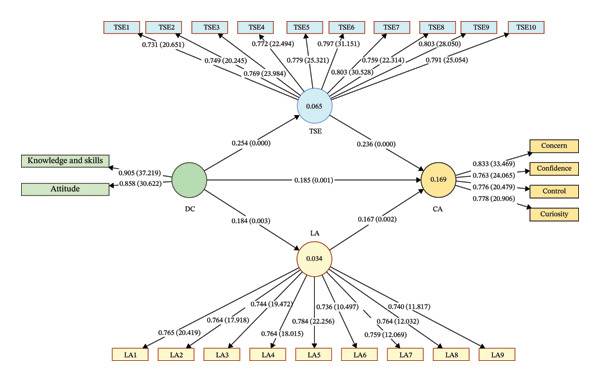
Structural model of the study. Note: Path coefficients and associated *p* values are presented along the direct paths. Outer loadings and corresponding *t*‐values for each indicator are displayed on the measurement model. DC: digital competence; TSE: technological self‐efficacy; LA: learning agility; CA: career adaptability. TSE and LA represent the mediating variables linking digital competence to career adaptability in the structural model.

Table 5 and Figure 1 present the bootstrapped estimates of direct, indirect, and total effects among the study constructs. Regarding direct effects, digital competence had a significant positive impact on career adaptability (*β* = 0.185, *t* = 3.441, *p* < 0.01; supporting H1), technological self‐efficacy (*β* = 0.254, *t* = 3.780, *p* < 0.001; supporting H2), and learning agility (*β* = 0.184, *t* = 3.001, *p* < 0.01; supporting H4). Additionally, both learning agility (*β* = 0.167, *t* = 3.067, *p* < 0.01) and technological self‐efficacy (*β* = 0.236, *t* = 3.998, *p* < 0.001) showed significant positive effects on career adaptability. The indirect effects of digital competence on career adaptability through technological self‐efficacy (*β* = 0.060, *t* = 2.593, *p* < 0.05; supporting H3) and learning agility (*β* = 0.031, *t* = 2.111, *p* < 0.05; supporting H5) were also significant, demonstrating partial mediation. The total indirect effect of digital competence on career adaptability via both mediators (technological self‐efficacy and learning agility) was significant (*β* = 0.091, *t* = 3.204, *p* < 0.01), suggesting that technological self‐efficacy and learning agility together play a meaningful role in explaining the relationship between digital competence and career adaptability. Finally, the total effect of digital competence on career adaptability, which accounts for both direct and indirect pathways, was substantial (*β* = 0.276, *t* = 4.997, *p* < 0.001), confirming the overall importance of digital competence in shaping career adaptability. The 95% confidence intervals for all effects did not include zero, further supporting the statistical significance of these relationships.

**TABLE 5:** Bootstrapped direct, indirect, and total effects in the structural equation model.

Coefficients paths	*β*	*T* statistics	95% CI	
			Lower	Upper
DC‐ > CA	0.185	3.441^∗∗^	0.080	0.287
DC‐ > LA	0.184	3.001^∗∗^	0.070	0.309
DC‐ > TSE	0.254	3.780^∗∗∗^	0.122	0.385
LA‐ > CA	0.167	3.067^∗∗^	0.061	0.274
TSE‐ > CA	0.236	3.998^∗∗∗^	0.118	0.351

*Indirect effect*				
DC‐ > TSE‐ > CA	0.060	2.593^∗^	0.021	0.112
DC‐ > LA‐ > CA	0.031	2.111^∗^	0.009	0.065
Total indirect effect	0.091	3.204^∗∗^	0.044	0.153
Total effect	0.276	4.997^∗∗∗^	0.168	0.383

Abbreviations: CA = career adaptability, DC = digital competence, LA = learning agility, TSE = technological self‐efficacy.

^∗^ *p* < 0.05; ^∗∗^ *p* < 0.01; ^∗∗∗^ *p* < 0.001.

## 5. Discussion

This study investigated the parallel mediating roles of technological self‐efficacy and learning agility in the relationship between digital competence and career adaptability among nurses.

The study found that digital competence positively predicted career adaptability, supporting H1. This finding indicates that nurses with higher levels of digital competence are more capable of adjusting to dynamic clinical environments, responding effectively to evolving healthcare technologies, and navigating complex professional transitions.

This result aligns with previous studies reporting that digital competence enhances professional adaptability and resilience among healthcare practitioners. For instance, Amin et al. [29] emphasized that digital competence facilitates lifelong learning and motivation among nursing students, while Longhini et al. [38] highlighted that digital health literacy enables healthcare professionals to adapt to organizational and technological transformations.

Consistent with H2, digital competence positively predicted technological self‐efficacy. This suggests that nurses with higher digital competence feel more confident in their ability to effectively utilize digital tools in clinical settings. Such confidence likely arises from prior successful experiences with digital systems, including electronic health records and telehealth technologies, reinforcing nurses’ perceptions of their technological capabilities. These findings align with Al‐Rahmi et al. [39] and Iraola‐Real et al. [40], who found a relationship between digital self‐efficacy and technology acceptance. National‐level evidence from Egypt also supports this relationship, showing a positive link between core competencies and self‐efficacy [41]. Differences across studies likely reflect variations in organizational, cultural, and contextual factors.

Consistent with H3, technological self‐efficacy was shown to mediate the relationship between digital competence and career adaptability. This indicates that digital skills alone are insufficient for enhancing career adaptability; it is the confidence in applying these skills that enables nurses to effectively navigate job demands and challenges. By mediating this relationship, technological self‐efficacy facilitates the translation of digital competence into improved professional performance and adaptive career behaviors. Early exposure to technology and continuous learning opportunities further confirms this mediating effect [26, 42]. Nurses confident in their technological abilities tend to demonstrate greater flexibility, resilience, and readiness in managing career transitions. This underscores self‐efficacy as a vital psychological resource for coping with occupational and technological changes. Supporting evidence includes Li et al. [42], who reported that nurses with higher technological self‐efficacy more effectively adjust to workplace changes and meet job demands, and Zhang et al. [1], who highlighted the role of early technology adoption and ongoing digital training in fostering both self‐efficacy and adaptability. From a theoretical perspective, this finding can be interpreted through the lens of career construction theory [43], which posits that adaptability represents an individual’s psychosocial resources for managing current and anticipated career tasks. Digital competence, as a domain‐specific capability, may strengthen these adaptive resources by enhancing self‐efficacy, problem‐solving ability, and openness to technological innovation.

In support of H4, digital competence was also positively associated with learning agility. This finding indicates that nurses who are more digitally competent are better able to adapt to new learning environments and leverage technology to acquire new knowledge and skills. Such adaptability is essential in the modern healthcare context, where technology‐driven changes are frequent. The results are consistent with Kim et al. [44], who reported a significant positive effect of digital competence on learning agility among higher education students, and with Nadzim et al. [31], who demonstrated that perceived digital competence enhances agility among employees in Malaysia. These findings highlight that digital skills not only improve task performance but also facilitate adaptive learning behaviors crucial for ongoing professional development.

The study found that learning agility mediated the relationship between digital competence and career adaptability, supporting H5. This result highlights the pivotal role of learning agility as a psychological mechanism through which digital competence translates into adaptive career behaviors among nurses. In essence, nurses who possess strong digital competence are not only proficient in using technology but also demonstrate a greater capacity to learn from experience, apply new knowledge in unfamiliar situations, and respond constructively to professional challenges. These learning‐oriented attributes enable them to leverage technological skills for continuous personal and professional growth, thereby enhancing their career adaptability.

Although no prior study has directly tested this specific mediation model in nursing staff, existing research supports the plausibility of these relationships. For example, Patwardhan et al. [30] showed that learning agility mediates the link between digital competence and perceived learning in higher education students. Ma and Yang [45] found a similar mediating role of learning agility between professional identity and job satisfaction among Chinese teachers. Other studies have also shown that learning agility mediates the relationship between job involvement and job satisfaction in educational and organizational settings. In nursing, Tawfik and El‐Fattah Mahgoub [46] highlighted that nurses’ learning agility plays a key mediating role between leadership and professional readiness. Building on this, the current study demonstrates that learning agility also mediates the relationship between digital competence and career adaptability, emphasizing its crucial role in turning nurses’ digital skills into practical adaptability and readiness for evolving career demands.

From a theoretical standpoint, this mediational pathway aligns with experiential learning theory [47], which suggests that learning is a process of transforming experiences into knowledge and action. Digitally competent nurses can more effectively engage in the full cycle of experiential learning that includes acquiring knowledge, reflecting on it, conceptualizing ideas, and applying them in practice, thereby enhancing their ability to adapt to new challenges. In this way, learning agility acts as a self‐regulatory process, helping nurses respond flexibly and effectively to technological and organizational changes. Importantly, this study is the first to show in a nursing context that learning agility serves as a bridge linking digital competence to career adaptability.

These findings gain particular relevance in the post‐COVID context, where digital technologies have reshaped nursing practice. The need for rapid adaptation to electronic health records, telehealth, and virtual learning platforms highlights the critical role of digital competence and learning agility in nursing. The observed mediating mechanisms underscore how nurses’ technological self‐efficacy and adaptive learning behaviors translate digital skills into practical career adaptability, reflecting unique challenges and opportunities in contemporary nursing practice.

Although digital competence, technological self‐efficacy, and learning agility collectively explained a modest proportion of variance in career adaptability (*R* ^2^ = 0.169), this level of explanatory power is not unusual in behavioral and organizational research, where complex outcomes are typically shaped by multiple individual and contextual factors. The present study intentionally focused on three key personal resources relevant to digital transformation in healthcare including digital competence, technological self‐efficacy, and learning agility. However, additional organizational and environmental factors may also play an important role in shaping nurses’ career adaptability. Future research could therefore extend the model by incorporating predictors such as perceived organizational support, leadership style, job demands, work environment characteristics, and access to professional development opportunities. Such factors may provide a more comprehensive understanding of the mechanisms that support nurses’ adaptability in rapidly evolving healthcare systems. Furthermore, although the observed effect sizes were relatively small, they remain theoretically meaningful, particularly in organizational settings where multiple variables collectively influence professional outcomes.

### 5.1. Limitations and Future Research

Several limitations should be acknowledged when interpreting the results of this study. First, the cross‐sectional design limits the ability to infer causal relationships among the study variables. Although the structural model specifies directional paths based on theoretical assumptions, the observed associations should be interpreted as relational rather than causal. Longitudinal or experimental research designs are therefore recommended to validate the temporal ordering and causal mechanisms underlying the relationships identified in this study.

Second, data were collected using self‐report questionnaires, which may introduce common method bias and increase the risk of inflated associations among variables measured from the same source. Although procedural remedies such as assuring participant anonymity and using validated measurement scales were applied to reduce this risk, future research could employ multiple data sources or time‐lagged data collection designs to further minimize potential common method variance.

Third, participants may have overestimated their digital competence or career‐related capabilities, introducing social desirability or response bias. Additionally, the sample was predominantly young, female, and early‐career nurses, recruited through convenience sampling from university hospitals in Egypt. Cultural, institutional, and technological factors in this context may limit the generalizability of the findings to other regions, healthcare settings, or more experienced nurse populations.

Fourth, although multigroup analyses (e.g., by gender or age) and inclusion of control variables such as years of experience could provide additional insights and potentially explain more variance in career adaptability, these analyses were beyond the scope of the present study due to the primary focus on testing the proposed mediation model. Future research with larger and more diverse samples is encouraged to explore potential subgroup differences and the impact of demographic or professional factors, which may enhance the explanatory power of the model and provide a more nuanced understanding of how digital competence translates into career adaptability across different nursing populations.

Future research should therefore replicate this model in diverse clinical and cultural contexts to determine whether similar relationships emerge across healthcare systems with varying levels of technological integration. Additionally, future studies could explore potential mediating or moderating variables, such as organizational support, digital leadership, or training opportunities, to deepen understanding of the pathways linking digital competence and career adaptability.

### 5.2. Implications of the Study

The findings of this study highlight digital competence as a fundamental component of modern nursing practice. As healthcare systems increasingly integrate digital technologies such as electronic health records, telehealth platforms, and clinical decision‐support systems, nurses must not only possess technical knowledge but also develop the technological self‐efficacy and learning agility required to adapt to rapidly evolving clinical environments. Strengthening these capabilities can enhance nurses’ confidence in using digital tools, thereby improving the quality, safety, and efficiency of patient care.

From a practical perspective, the results provide important guidance for the design of nursing training and professional development programs. Educational institutions and healthcare organizations should incorporate structured digital training modules within undergraduate curricula, internship programs, and continuing professional education. For example, simulation‐based workshops, hands‐on training sessions in electronic health record systems, and digital health technology laboratories may enhance nurses’ technological self‐efficacy by providing experiential learning opportunities. Mentorship programs and peer‐assisted learning approaches may also facilitate the development of learning agility, enabling nurses to adapt more effectively to new technologies and clinical innovations.

Additionally, targeted professional development initiatives such as digital competency workshops, short certification courses in health informatics, and interdisciplinary training programs involving information technology specialists can support nurses in translating digital knowledge into clinical practice. These interventions may strengthen both digital competence and technological self‐efficacy, which in turn can improve nurses’ preparedness to engage with digital healthcare systems.

At the policy level, healthcare leaders and educational policymakers should view digital competence development as a strategic investment in workforce sustainability. Institutional policies should support continuous digital skills training, provide access to technological resources, and encourage lifelong learning among nurses. Integrating digital competence and adaptive learning capabilities into national nursing competency frameworks, accreditation standards, and workforce development strategies may contribute to building a resilient nursing workforce capable of delivering safe, innovative, and technology‐enabled care in the digital era.

## 6. Conclusions

This study provides empirical evidence that nurses’ digital competence is positively associated with their career adaptability in increasingly technology‐oriented healthcare systems. The findings suggest that digital competence is linked to higher levels of career adaptability, both directly and indirectly through technological self‐efficacy and learning agility.

These results indicate that nurses who report stronger digital capabilities also tend to demonstrate greater confidence in using technology and higher learning agility, which are in turn related to greater adaptability in their professional roles. Together, these factors highlight the multifaceted nature of professional development in contemporary nursing environments, where technological competence, psychological confidence, and adaptive learning capabilities appear to function in a complementary manner.

## Author Contributions

Ibrahim Abdullatif Ibrahim: conceptualization; methodology; formal analysis; investigation; data curation; writing–original draft; and visualization. Samar Atef Mounib: methodology; investigation; data curation; and writing–review and editing. Eman Saad Eldesoky: investigation; data curation; and writing–review and editing. Reda Shehata Elsayed: methodology; validation; writing–review and editing; and supervision. Mennat Allah G. Abou Zeid: conceptualization; supervision; project administration; and writing–review and editing. Hala Gaber Elatroush: investigation; resources; and writing–review and editing.

## Funding

No funds, grants, or other support was received.

## Disclosure

All authors have read and approved the final version of the manuscript and agree to be accountable for all aspects of the work.

## Conflicts of Interest

The authors declare no conflicts of interest.

## Supporting Information

Table 1s. Full List of Measurement Scale Items and Dimensions. Table 2s. Assessment is a reflective–reflective higher‐order construct of the study (digital competence and career adaptability). Table 3s. Discriminant validity of the constructs. Table 4s. Descriptive statistics of the study variables.

## Supporting information


**Supporting Information** Additional supporting information can be found online in the Supporting Information section.

## Data Availability

The data that support the findings of this study are available on request from the corresponding author. The data are not publicly available due to privacy or ethical restrictions.
