# Correction to “Digital Competence and Career Adaptability Among Nurses: The Parallel Mediating Roles of Technological Self‐Efficacy and Learning Agility”

**DOI:** 10.1155/jonm/9838523

**Published:** 2026-09-24

I. A. Ibrahim, S. A. Mounib, E. S. Eldesoky, R. S. Elsayed, M. A. G. Abou Zeid, and H. G. Elatroush, “Digital Competence and Career Adaptability Among Nurses: The Parallel Mediating Roles of Technological Self‐Efficacy and Learning Agility,” *Journal of Nursing Management* 2026 (2026): 7770229, https://doi.org/10.1155/jonm/7770229.

In the published version of this article, the numbering of affiliations for some authors was incorrect.

The affiliation number for Eman Saad Eldesoky was published as ^4^ and should be corrected to ^3^.

For Reda Shehata Elsayed, affiliation ^4^ was omitted and should be added alongside affiliation ^2^.

The correct author and affiliation numbering should read as follows:

Ibrahim Abdullatif Ibrahim^1,2^, Samar Atef Mounib^3^, Eman Saad Eldesoky^3^, Reda Shehata Elsayed^2,4^, Mennat Allah G. Abou Zeid^5,6^, and Hala Gaber Elatroush^2^.

We apologize for this error.

