# Supplementary material for: Digital Competence and Career Adaptability Among Nurses: The Parallel Mediating Roles of Technological Self‐Efficacy and Learning Agility
Source: J Nurs Manag. 2026 Apr 17;2026:7770229. doi: 10.1155/jonm/7770229 (PMC13088261; doi:10.1155/jonm/7770229)
Supplement: Supplementary file 1 — Supporting Information Additional supporting information can be found online in the Supporting Information section. [file JONM-2026-7770229-s001.docx]

| Table 1s. Full List of Measurement Scale Items and Dimensions |
| --- |
| 1. **Digital Competence Scale** |
| **Dimension 1: Knowledge & Skills** |
| 1. I am familiar with digital technologies at my workplace. |
| 1. I feel confident about using digital technology to find relevant information. |
| 1. I feel confident about using digital technology to communicate. |
| 1. I feel confident about using digital technology to obtain data and information on clinical care. |
| 1. I am able to reach conclusions based on information acquired through digital technologies. |
| 1. I feel confident in dealing with confidentiality issues relating to digital technology at my workplace. |
| **Dimension 2: Attitude** |
| 1. Digital technology fits well with the way I like to work. |
| 1. I enjoy using digital technology at my workplace. |
| 1. I like to use digital technology at work. |
| 1. I believe that digital technology provides numerous benefits in terms of quality of care. |
| 1. I believe that digital technology improves patient outcomes. |
| 1. I believe that digital technology is beneficial for my patients. |
| 1. **Technological self-efficacy** |
| 1. Acquire new knowledge in technology in a short time, considering that many technologies change rapidly. |
| 1. Finding answers to challenging situations that arise from my work practice using search engines and applications that use the internet (web-based). |
| 1. Gain new knowledge by following full-online courses or seminars. |
| 1. Become familiar with new applications or technologies, preferring learning strategies focused on practical use rather than on “taking notes”. |
| 1. Plan a self-learning curve to improve my digital and technological skills. |
| 1. Send an e-mail containing numerous file attachments. |
| 1. Start a video call within minutes with the tools I have at my disposal. |
| 1. Stay up-to-date with new versions of applications, digital services, and devices. |
| 1. Effectively collaborate in teams while also utilizing digital tools (example, video calls, e-mail, etc.). |
| 1. Overcoming any preconception about the tech world (example, effectiveness of distance learning). |
| 1. **Learning Agility Scale** |
| 1. Is curious and inquisitive. |
| 1. Accepts and acts on feedback from others. |
| 1. Is flexible; adjusts his/her approach when something doesn't work. |
| 1. Is self-aware; knows own strengths and limitations. |
| 1. Displays a desire to gain new knowledge and skills. |
| 1. Actively pursues personal growth and improvement. |
| 1. Seeks out challenges and new experiences. |
| 1. Is open-minded and receptive to change and new ideas. |
| 1. Reflects on and learns from mistakes. |
| 1. **Career Adapt-Abilities Scale-Short Form** |
| **Dimension1: Concern** |
| 1. Thinking about what my future will be like. |
| 1. Preparing for the future. |
| 1. Becoming aware of the educational and vocational choices that I must make. |
| **Dimension2: Control** |
| 1. Making decisions by myself. |
| 1. Taking responsibility for my actions. |
| 1. Counting on myself. |
| **Dimension3: Curiosity** |
| 1. Looking for opportunities to grow as a person. |
| 1. Investigating options before making a choice. |
| 1. Observing different ways of doing things. |
| **Dimension4: Confidence** |
| 1. Taking care to do things well. |
| 1. Learning new skills. |
| 1. Working up to my ability. |

Table 2s. Assessment reflective-reflective higher order construct of the study (Digital competence and career adaptability)

| Constructs | Dimensions | Items | Loading | VIF | Reliability | | Convergent validity |
| --- | --- | --- | --- | --- | --- | --- | --- |
|  |  |  |  |  | α | CR | AVE |
| Digital competence  (DC) | Knowledge and skills | DC1 | 0.705 | 1.516 | 0.848 | 0.887 | 0.567 |
|  |  | DC2 | 0.782 | 1.794 |  |  |  |
|  |  | DC3 | 0.794 | 2.088 |  |  |  |
|  |  | DC4 | 0.752 | 1.847 |  |  |  |
|  |  | DC5 | 0.733 | 2.077 | 0.881 | 0.908 | 0.622 |
|  |  | DC6 | 0.749 | 2.045 |  |  |  |
|  | Attitude | DC7 | 0.730 | 2.047 |  |  |  |
|  |  | DC8 | 0.822 | 2.495 |  |  |  |
|  |  | DC9 | 0.792 | 2.363 |  |  |  |
|  |  | DC10 | 0.794 | 2.339 |  |  |  |
|  |  | DC11 | 0.820 | 2.249 |  |  |  |
|  |  | DC12 | 0.770 | 1.869 |  |  |  |
| Career adaptability  (CA) | Concern | CA1 | 0.821 | 1.846 | 0.755 | 0.859 | 0.67 |
|  |  | CA2 | 0.806 | 2.018 |  |  |  |
|  |  | CA3 | 0.828 | 1.810 |  |  |  |
|  | Control | CA4 | 0.790 | 1.906 | 0.767 | 0.864 | 0.679 |
|  |  | CA5 | 0.825 | 2.027 |  |  |  |
|  |  | CA6 | 0.856 | 2.440 |  |  |  |
|  | Confidence | CA7 | 0.850 | 2.503 | 0.836 | 0.901 | 0.752 |
|  |  | CA8 | 0.904 | 2.503 |  |  |  |
|  |  | CA9 | 0.849 | 2.251 | 0.836 | 0.901 | 0.753 |
|  | Curiosity | CA10 | 0.855 | 1.611 |  |  |  |
|  |  | CA11 | 0.907 | 1.574 |  |  |  |
|  |  | CA12 | 0.839 | 1.421 |  |  |  |

Table 3s. Discriminant validity of the constructs

| HTMT | Knowledge and skills | Attitude | Concern | Control | Confidence | Curiosity |
| --- | --- | --- | --- | --- | --- | --- |
| Knowledge and skills |  |  |  |  |  |  |
| Attitude | 0.646 |  |  |  |  |  |
| Concern | 0.300 | 0.172 |  |  |  |  |
| Control | 0.204 | 0.187 | 0.736 |  |  |  |
| Confidence | 0.194 | 0.221 | 0.611 | 0.503 |  |  |
| Curiosity | 0.231 | 0.244 | 0.582 | 0.714 | 0.628 |  |
| Fornell-Larcker |  |  |  |  |  |  |
| Knowledge and skills | 0.753 |  |  |  |  |  |
| Attitude | 0.558 | 0.789 |  |  |  |  |
| Concern | 0.245 | 0.154 | 0.818 |  |  |  |
| Control | 0.171 | 0.163 | 0.542 | 0.824 |  |  |
| Confidence | 0.173 | 0.205 | 0.491 | 0.403 | 0.867 |  |
| Curiosity | 0.199 | 0.217 | 0.457 | 0.578 | 0.528 | 0.868 |

Table 4s. Descriptive statistics of the study variables

| Variables of the study | Minimum | Maximum | Mean (SD) |
| --- | --- | --- | --- |
| Digital competence | 1.92 | 4.33 | 3.46 (0.42) |
| Knowledge and skills | 1.67 | 4.33 | 3.50 (0.44) |
| Attitude | 1.33 | 4.67 | 3.42 (0.51) |
| Technological self-efficacy | 1.40 | 4.80 | 3.64 (0.46) |
| Learning agility | 2.00 | 4.89 | 3.85 (0.47) |
| Career adaptability | 3.33 | 5.00 | 4.50 (0.43) |
| Concern | 2.00 | 5.00 | 4.47 (0.38) |
| Control | 3.00 | 5.00 | 4.53 (0.46) |
| Confidence | 3.00 | 5.00 | 4.52 (0.48) |
| Curiosity | 3.00 | 5.00 | 4.47 (0.49) |
